# Targeting of acute myeloid leukemia by five-gene engineered T cells expressing transgenic T-cell receptor specific to WT1, chimeric antigenic receptor specific to GM-CSF receptor, bispecific T-cell engager specific to CD33, and tEGFR suicide gene system

**DOI:** 10.1093/immadv/ltaf022

**Published:** 2025-06-11

**Authors:** Kristýna Šmilauerová, Martin Štach, Martin Mucha, Šárka Vaníková, Jana Rychlá, Pavel Otáhal

**Affiliations:** Institute of Hematology and Blood Transfusion, Department of Immunotherapy, Prague, Czechia; Faculty of Natural Sciences, Charles University, Prague, Czechia; Institute of Hematology and Blood Transfusion, Department of Immunotherapy, Prague, Czechia; Faculty of Natural Sciences, Charles University, Prague, Czechia; Institute of Hematology and Blood Transfusion, Department of Immunotherapy, Prague, Czechia; Faculty of Natural Sciences, Charles University, Prague, Czechia; Institute of Hematology and Blood Transfusion, Department of Immunotherapy, Prague, Czechia; Institute of Hematology and Blood Transfusion, Department of Immunomonitoring and flow cytometry, Prague, Czechia; Institute of Hematology and Blood Transfusion, Department of Immunotherapy, Prague, Czechia; Institute of Hematology and Blood Transfusion, Department of Immunotherapy, Prague, Czechia

**Keywords:** transgenic T-cell receptor, chimeric antigen receptor, WT1, acute myeloid leukemia, piggyBac transposon

## Abstract

**Background:**

Cancer immunotherapy with transgenic T-cell receptor-engineered T cells (TCR-T) enables the targeting of intracellular tumor-specific antigens; in contrast, chimeric antigen receptor-modified T cells (CAR-T) mediate tumor cell killing via the recognition of surface antigens. In the case of acute myeloid leukemia, the lack of leukemia-specific surface antigens limits the efficacy of CAR-T cells; therefore, TCR-T cells may represent a more targeted immunotherapy approach. However, the tumor immunosuppressive environment eliminates the best-functioning, high-avidity TCR-T cells, thus creating a need for novel, enhanced TCR-T cells.

**Methods:**

The piggyBac transposon vector used for gene modification of T cells expresses a T-cell receptor specific to the WT1 tumour antigen, an NFAT promoter-regulated CAR specific to GM-CSF receptor, a CD3xCD33 bispecific T-cell engager, and a truncated EGFR suicide gene system. The transgenic T cells were generated by electroporation using a single expression vector, and the efficiency of these engineered TCR-T cells was evaluated using models that utilized AML cell lines and primary AML cells.

**Results:**

The NFAT-driven GM-CSF CAR significantly enhances the antileukemic activity of WT1-specific TCR-T cells, which importantly maintain specificity for their HLA/peptide antigenic complex. Next, by inserting the CD3xCD33 bispecific T-cell engager into the transposon vector, both TCR-T cells and recruited non-transfected bystander T cells can efficiently target the CD33 antigen, providing more robust antileukemic effects.

**Conclusion:**

The presented strategy, utilizing a single piggyBac transposon vector, enables the complex redirection of T-cell specificity against acute myeloid leukemia by inserting TCR, CAR, BiTE constructs, along with a tEGFR gene suicide system.

## Graphical Abstract

**Figure:**
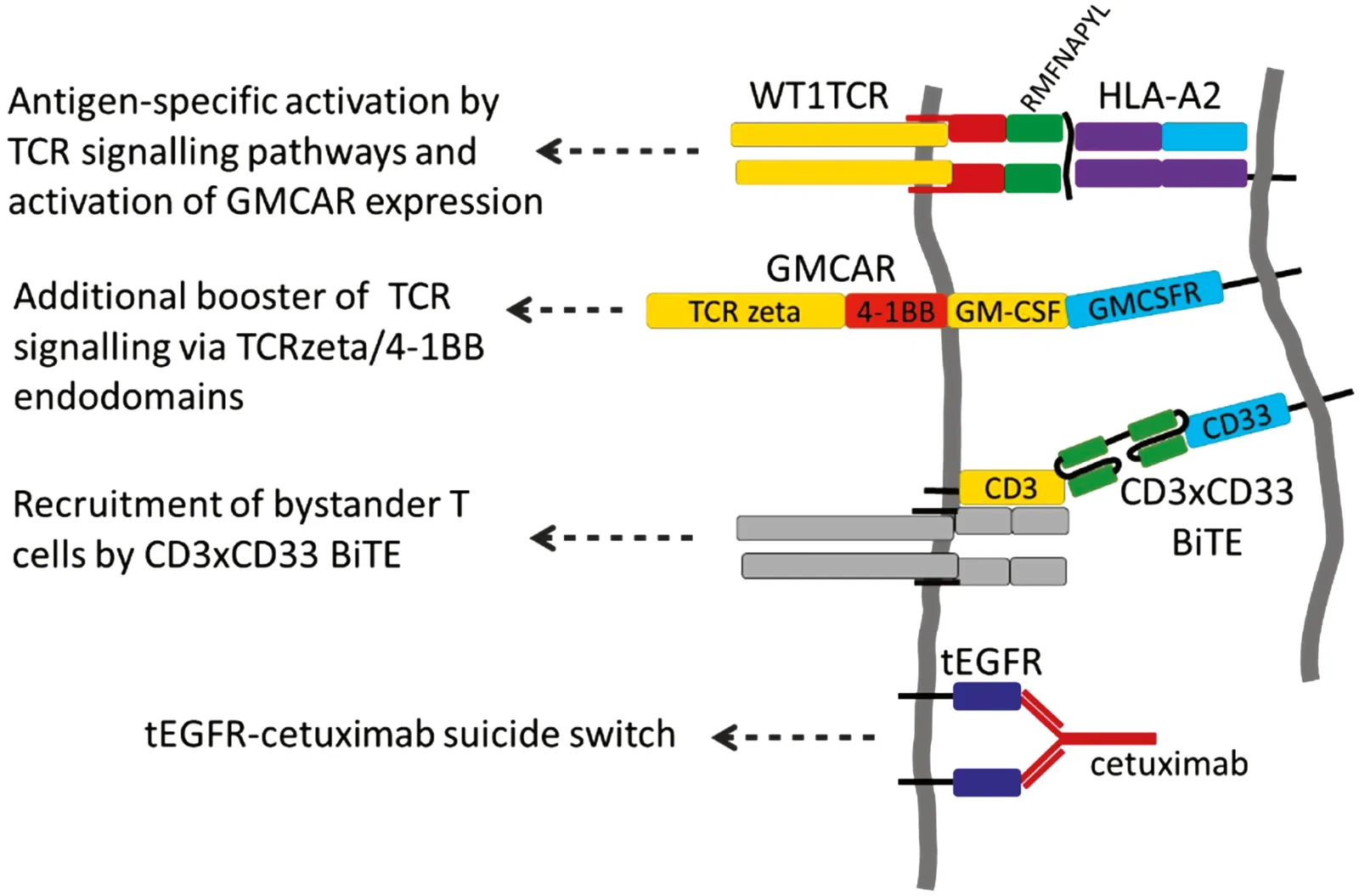
Herein, we engineered T cells with piggyBac transposon expressing a T-cell receptor specific to the WT1 tumor antigen (WT1TCR), an NFAT-regulated CAR specific to GM-CSF receptor (GMCAR), a CD3xCD33 bispecific T-cell engager (BiTE), and a truncated EGFR (tEGFR) suicide gene system. Our findings show that the NFAT-driven GMCAR significantly enhances the antileukemic functions of WT1TCR T cells without suppressing the specificity of TCR-T cells using models utilizing AML cell lines and primary AML cells. By inserting the BiTE into the transposon, both TCR-T cells and recruited nontransfected bystander T cells target the CD33 antigen, resulting in a more robust antileukemic effect, albeit at the cost of myelotoxicity.

## Introduction

Cancer immunotherapy with gene-engineered T cells by introducing recombinant T-cell receptors (TCRs) or chimeric antigen receptors (CARs) has proven its efficiency in the last decade. Combinatorial targeting of multiple antigens enables specific recognition of malignant cells while preserving healthy cells co-expressing the tumor-specific antigens and reducing the risk of on-target off-cancer toxicity. In the case of acute myeloid leukemia (AML), the efficiency of cellular immunotherapy is limited by the lack of proper surface antigens enabling selective targeting of AML cells by CARs [1]. On the other hand, targeting intracellular AML antigens such as WT1 by TCRs provides potent specificity but is limited by the diversity of HLA among patients. Although the adoptive T-cell therapies via transgenic T-cell receptor-expressing T cells (TCR-T) enable selective targeting of intracellular tumor-specific antigens in an HLA-dependent manner, the affinity and avidity of the T-cell receptor primarily define the antitumor response in T-cell immunotherapy [2]. Unfortunately, the best-functioning high-avidity TCRs are often either eliminated by immune tolerance mechanisms or inhibited by tumor escape strategies [3]. These can be detected among TILs or in peripheral blood expressing an exhausted phenotype [4, 5]. Low-avidity TCRs cloned from tumor-reactive T cells isolated from cancer patients can be engineered in vitro to increase their avidity for antigens; alternatively, high-avidity TCRs can be identified from healthy donors in the case of the most frequent HLA alleles, such as A2 [6, 7]. However, artificially engineered high-avidity TCR-T cells might acquire specificity to different antigens and cause off-target toxicities [7, 8].

Here, we focused on complex gene engineering of TCR-T cells with specificity for Wilms’ Tumor 1 (WT1), a tumor antigen overexpressed by a wide range of hematological and solid tumors [9]. We constructed a piggyBac transposon vector containing three promoters driving the expression of five separate proteins. An NFAT-inducible promoter regulates the first expression cassette—it produces a GMCAR, a ligand-based CAR comprising a GM-CSF (granulocyte-macrophage colony-stimulating factor) as an antigen recognition site, CD8 hinge and transmembrane region, 4-1BB co-stimulatory domain, and CD3ζ chain. The GMCAR targets the GM-CSF receptor α chain (CD116), expressed by normal monocytic lineage cells and overexpressed in 60%-80% of AML cases. The second cassette is regulated by a ubiquitin promoter (UBC) and produces a recombinant HLA-A*0201+ (HLA-A2)-restricted WT1-specific TCR alpha and beta chains and a truncated EGFR (tEGFR) surface antigen, separated by P2A and T2A motifs, respectively. Finally, a CMV promoter drives the third cassette and produces a secretable bispecific T-cell engager (BiTE) antibody specific for a myeloid antigen CD33. The goals were to enhance the WT1-specific TCR activation by inducibly expressed GMCAR, to provide an EGFR-cetuximab suicide gene system, and to enable the recruitment of natural nonmodified bystander T cells via secretable CD3xCD33 BiTE.

There have been similar efforts to combine CAR and TCR in various ways. The simultaneous modification of cells by both CAR and TCR has been reported to be successful in multiple studies [10, 11]. Other approaches use CAR functioning as a co-stimulatory signal for TCR activation [12], CAR employing a TCR-like antibody [13], or CAR combined with a bispecific antibody [14, 15]. Our approach is novel due to the combination of all three molecules, with the CAR being expressed only upon TCR-mediated activation.

The transposon was transfected into T cell via electroporation followed by anti-CD3/CD28 stimulation and expansion in the presence of cytokines IL-7 and IL-15, similarly as described previously [16]. The biological activity of the generated TCR-T cells was determined by in vitro studies using AML cell lines THP-1 and K562 and with primary AML cells obtained from patients. Furthermore, the safety profile of the produced TCR-T cells on normal hematopoiesis was determined by bone marrow (BM) cellular co-culture experiments.

The NFAT GMCAR construct significantly enhanced the cytotoxicity of the WT1-specific TCR against AML cell lines as well as against primary AML cells. The recognition of the WT1 antigen by the transgenic TCR activated the NFAT-responsive promoter and increased the expression of the GMCAR, which further enhanced the T-cell activation via the recognition of its target—the GM-CSF receptor—altogether, resulting in enhanced proliferation and persistence of their effector functions. Furthermore, we observed that the cytotoxicity of the NFAT GMCAR WT1TCR T cells was highly sensitive to cyclosporin (CsA). In contrast, T cells constitutively expressing GMCAR under UBC promoter (UBC GMCAR T cells) could not be suppressed by CsA at therapeutical concentrations—it is known that CAR T cells are generally insensitive to common immunosuppressants [17]. In the next step, the NFAT GMCAR WT1TCR transposon was engineered to co-express a CD3xCD33 BiTE to generate five-gene modified T cells (designated as 5G WT1TCR-T cells). Our results interestingly revealed that the BiTE further enhanced the antileukemic potency of engineered T cells against AML cell lines and primary AML cells by recruiting nontransfected (NT) bystander T cells.

In summary, the presented mechanisms of combined co-expression of CAR and TCR transgenes might represent a feasible approach to increase the efficacy of TCR-based therapies.

## Results

### Inducibly expressed GMCAR enhances the expansion of WT1TCR-T cells

**Figure 1.**
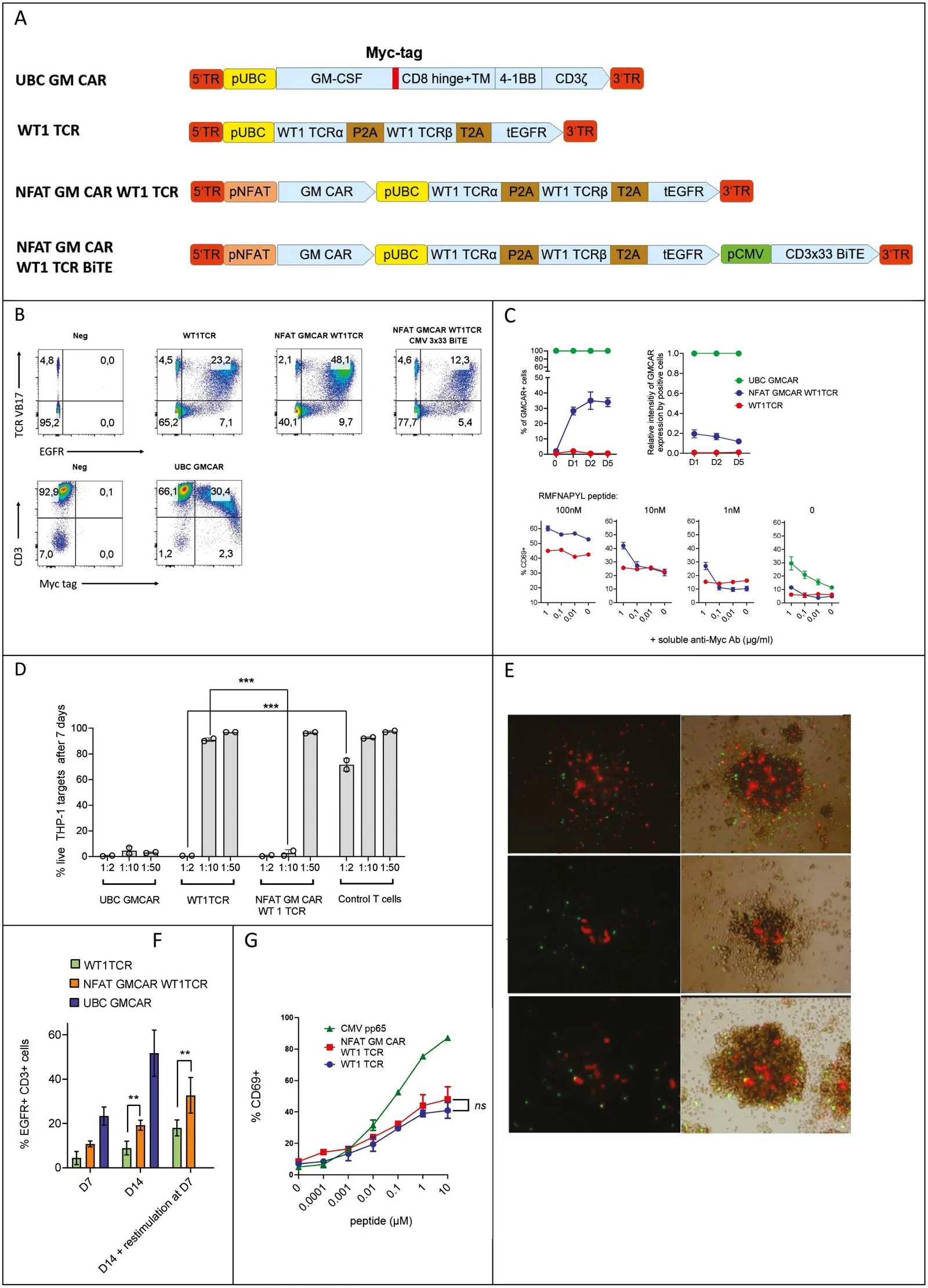
NFAT-regulated expression of GMCAR promotes TCR-dependent activation, proliferation, and effector functions of engineered TCR-T cells. (A) The design of transposon constructs. 5’TR / 3’ TR—terminal repeats on either end of the transposon, pUBC—ubiquitin C promoter, pNFAT—Nuclear factor of activated T-cell promoter, pCMV—cytomegalovirus promoter, P2A/T2 A—sequences for self-cleaving peptides, GM CAR—abbreviation for the first described construct (expressing membrane-bound GM-CSF, myc tag, CD8-derived hinge and transmembrane domain, 4-1 BB costimulation domain, CD3ζ chain), tEGFR—sequence for truncated EGFR molecule, CD3x33 BiTE—sequence for bispecific antibody targeting both CD3 and CD33. (B) The expression of the GM CAR, the transgenic TCR by WT1TCR, NFAT GMCAR WT1TCR, and NFAT GM CAR WT1TCR CMV 3x33 BiTE modified T cells. (C) The kinetics of NFAT GMCAR expression upon stimulation. UBC GMCAR T cells (top one when three lines are present) are shown here as a positive control. (D) NFAT GMCAR T cells have enhanced effector functions during long-term co-cultivation with THP-1 cells compared to WT1TCR T cells. (E) Visualization of the interaction between the TCR-T cells and AML targets. WT1TCR and NFAT GMCAR WT1TCR were co-transfected with GFP or RFP-expressing transposons, respectively. Visualized by fluorescent microscopy; one representative experiment out of three is presented. (F) NFAT GMCAR supports the expansion of the transfected T cells compared to WT1TCR T cells (*n* = 3, two-sided paired *t*-test was used to compare the groups). For comparison, UBC GMCAR T cells are shown in the graph. (G) WT1TCR T cells and NFAT GMCAR WT1TCR T cells have a similar sensitivity to the antigenic stimulation via the transgenic TCR. Two-sided paired *t*-test, *n* = 2.

The designs of the transposon constructs are presented in Fig. 1A. The expression of the transgenic TCR by WT1TCR T cells, NFAT GMCAR WT1TCR T cells, and NFAT GM CAR WT1TCR CMV 3x33 BiTE T cells was determined with anti-TCR Vβ17 and anti-EGFR antibodies on CD3+ T cells. Similarly, the expression of the GMCAR construct was measured with anti-Myc Ab on CD3+ cells. The expression of the individual transgenes is presented in Fig. 1B (the expression of the CD3x33 BiTE is further presented in Fig. 4A and B). The strength of the NFAT promoter was measured by determining the expression level of the GMCAR construct after activation with THP-1 cells and was compared to constitutively expressed UBC GMCAR construct (Fig. 1C). Like previously, the GMCAR construct was detected using an anti-Myc tag antibody on EGFR+CD8+CD3+ NFAT GMCAR WT1TCR T cells and WT1TCR T cells. The NFAT promoter increased the expression of GMCAR after antigenic stimulation on the fraction of modified T cells (approximately 32%), and the expression level was approximately five-fold lower compared to the constitutive UBC GMCAR construct. Next, we studied the effects of combined GMCAR and WT1TCR signaling using soluble anti-Myc Ab recognizing the GMCAR and with RMFNAPYL peptide specific to WT1 TCR-T cells produced from HLA A2 positive donors. We observed that GMCAR activation increased the expression of CD69 on modified T cells, thereby enabling intensified antigen-specific activation. The effects of NFAT GMCAR on the cytotoxicity of engineered T cells were tested by long-term (7 days) co-culture assays with THP-1 cells at a high excess of the target cells (E:T = 1:2, 1:10, and 1:50) (Fig. 1D). The remaining percentages of live THP-1 cells out of all live cells were measured by FACS using DAPI dye, anti-CD3 Ab, and FSC/SSC parameters. The main point was to identify the E:T ratio for each type of T cells, leading to the complete elimination of all THP-1 targets during the long-term co-cultivation. The results showed that NFAT GMCAR WT1TCR T cells were significantly more effective in eliminating THP-1 cells at an E:T ratio of 1:10 compared to WT1TCR T cells. At the same time, UBC GMCAR T cells overcame both types of TCR constructs and eliminated all THP-1 targets at E:T ratio of 1:50. The positive effects of the NFAT GMCAR are further demonstrated by an increased percentage of transfected EGFR+ T cells during the manufacturing. The percentages of the transfected T cells were measured 7 and 14 days post-electroporation, either without restimulation or, after restimulation at Day 7 with TCR Vβ17-specific Ab (Fig. 1F). Next, the WT1TCR and NFAT GMCAR WT1TCR T cells (all HLA A2 positive) were activated with RMFNAPYL peptide at decreasing concentration and, after 24 hours, were stained with anti-CD69 Ab to measure its upregulation among CD3+CD8+EGFR+ T cells. No significant differences in the efficiency of RMFNAPYL peptide-based activation between the NFAT GMCAR WT1TCR and the WT1TCR T cells were found (Fig. 1G). For comparison, similarly manufactured TCR-T cells specific for the CMV pp65/HLA-A2 antigenic complex were analogically activated with its specific peptide—this experiment further revealed that the WT1TCR construct has lower functional avidity than a CMV pp65-specific TCR—it is an expected result as the CMV pp65-specific TCR represents a highly functional virus-specific TCR.

To visualize the cytotoxicity, the NFAT GMCAR WT1TCR T cells and the WT1TCR T cells were fluorescently labeled by co-transfection with RFP and GFP-expressing transposons, respectively. Both types of T cells were mixed at a 1:1 ratio and were co-cultured with THP-1 cells. Images of live clusters of T cells and THP-1 cells were captured on Day 7 using fluorescent microscopy. While the WT1TCR T cells (GFP+) failed to expand effectively, the NFAT GMCAR WT1TCR T cells (RFP+) persisted and were localized within the centers of the THP-1 cell clusters (Fig. 1E).

### NFAT GMCAR WT1TCR T cells are HLA-restricted and are sensitive to cyclosporine

**Figure 2.**
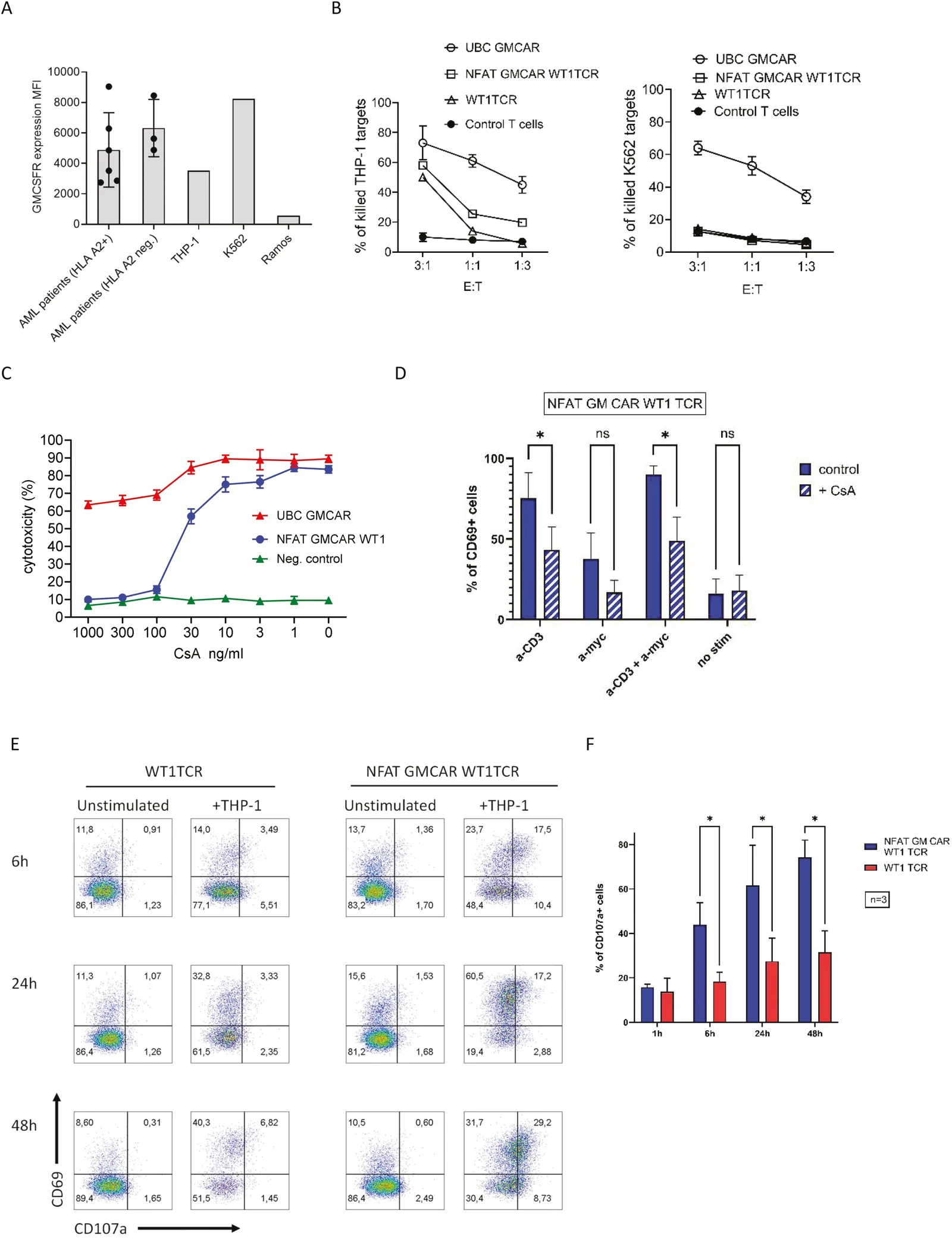
NFAT GMCAR WT1TCR T cells mediate killing in an HLA-dependent manner and are sensitive to cyclosporine. (A) The expression of the GMCSF receptor (GMCSFR) was measured with anti-GMCSFR Ab on AML blasts obtained from patients with newly diagnosed acute myeloid leukemia (HLA A2 positive, *n* = 6 and HLA A2 negative, *n* = 3) on myeloid cell lines THP-1, K562, and on B-cell line Ramos. (B) Cytotoxicity of the engineered T cells against THP-1 cells (HLA A2+WT1+ GMCSFR+) and K562 cells (HLA A2-GMCSFR+), *n* = 3. (C) Cyclosporine (CsA) effectively inhibits the cytotoxicity of NFAT GMCAR WT1TCR T cells; *n* =2. (D) CsA inhibits antigenic activation of NFAT GMCAR WT1TCR T cells following stimulation with anti-Myc tag and anti-CD3 plate-bound antibodies. Multiple *t*-tests between individual activation types, *n* = 3. (E and F) NFAT GMCAR WT1TCR T cells have a rapid onset of cytotoxicity compared to WT1TCR T cells. One representative experiment is presented (E). Comparison of activation and degranulation of the entire data sets (F), paired *t*-test, *n* = 3.

The ability of NFAT GMCAR WT1TCR T cells to kill AML targets via GM-CSF receptor, in addition to the RMFNAPYL/HLA-A2 antigenic complex, was further studied by cytotoxic test using THP-1 cells (HLA A2 positive) and K562 cells (HLA A2 negative) (Fig. 2B). The measured expression level of the GM-CSF receptor was higher on K562 cells than on THP-1 cells and is similar to its expression by primary AML blasts, which were used in subsequent experiments (Fig. 2A). The target cells were labeled with CFSE dye and co-cultured at indicated E:T ratios for 16 hours; the percentages of killed targets were determined by FACS via labeling with DAPI dye. Although the UBC GMCAR T cells effectively killed both THP-1 and K562 cells, the NFAT GMCAR WT1TCR T cells and the WT1TCR T cells killed only THP-1 cells indicating that the inducibly expressed GMCAR construct does not effectively trigger cytotoxicity via the GMCSF-GMCSFR interaction (Fig. 2B). In the next step, we focused on studying the sensitivity of the NFAT GMCAR WT1TCR T cells to pharmacological immunosuppression because it might represent an important safety measure from a clinical perspective. THP-1 targets were labeled with green fluorescent CFSE dye and co-cultured with indicated T cells at E:T = 1 in the presence of increasing concentration of CsA. The percentages of killed targets were measured 24 hours later. We found that cyclosporin A (CsA) effectively inhibits the cytotoxic activity of the NFAT GMCAR WT1TCR T cells at pharmacological levels. In contrast, the UBC GMCAR T cells could not be effectively suppressed by CsA (Fig. 2C). This finding was further confirmed by measuring the upregulation of CD69 after stimulation with anti-CD3 and anti-Myc tag antibodies in the presence or absence of CsA (Fig. 2D). First, in the absence of CsA the stimulation of the NFAT GMCAR WT1TCR T cells with immobilized anti-CD3 or, anti-Myc Abs, or both anti-Myc + anti-CD3 Abs activated 67.2%, 51.5%, and 86.5% of CD3+CD8+EGFR+ T cells, respectively (20.2% was the baseline in nonactivated T cells), further demonstrating the functionality of the NFAT GMCAR transgene in promoting the TCR-dependent activation. Next, in the presence of the CsA (200 ng/ml), only 36.6% (anti-CD3 Ab), 17.3% (anti-Myc Ab), and 47.8% (anti-Myc + anti-CD3 Abs) of CD3+CD8+EGFR+ T cells became activated (23.7% was the baseline). In the following, we measured the kinetics of the activation and cytotoxicity during co-culture with THP-1 cells by determining the level of degranulation and expression of CD69 at indicated time points (Fig. 2E and F). The outcome of this experiment demonstrated faster and stronger degranulation of NFAT GMCAR WT1TCR T cells compared to WT1 TCR-T cells (7% vs. 25% at 6 hours, 3% vs. 17% at 24 hours, 6% vs. 35% at 48 hours). In conclusion, NFAT GMCAR WT1TCR-T cells kill target cells in an HLA-dependent manner with faster onset than WT1TCR-T cells. Furthermore, CsA can effectively suppress their cytotoxicity at concentrations within the therapeutic range.

### NFAT GMCAR WT1 TCR-T cells have enhanced cytotoxicity against primary AML cells

**Figure 3.**
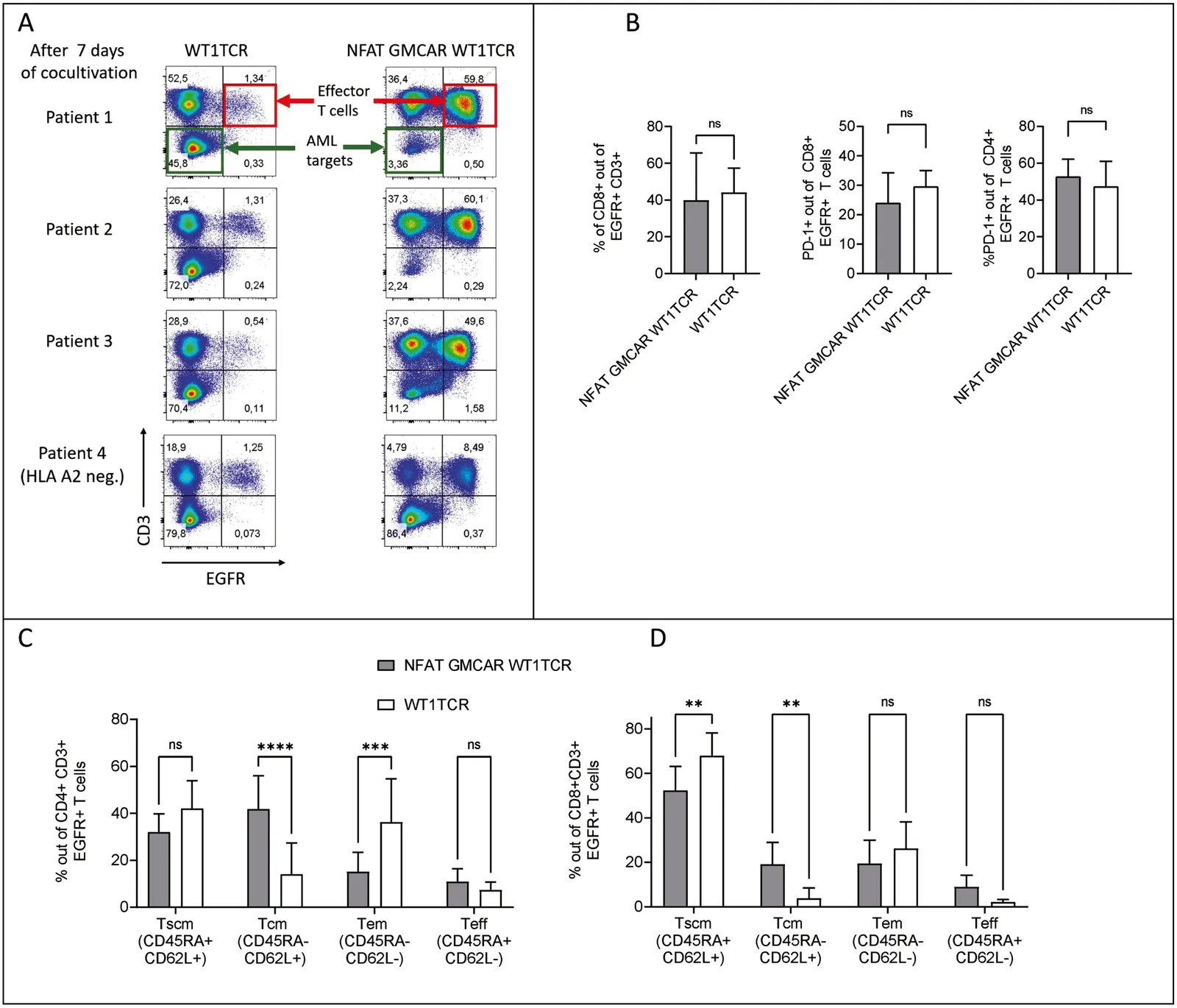
NFAT GMCAR transgene enhances the antileukemic function of WT1 TCR T cells against primary AML cells. (A) The representative dot plots of samples of three HLA A2+ and of one HLA A2-patients are presented. (B–D) We determined the outcome of antigenic stimulation with AML blasts based on the immunophenotype of engineered TCR-T cells.

We further studied the effects of the inducibly expressed GMCAR using the primary AML cells as antigenic targets. We obtained the AML cells via leukodepletion from newly diagnosed AML patients. These patients had high blast counts in the peripheral blood and were WT1-positive. Their level of GM-CSF receptor expression was comparable to AML cell lines THP-1 and K562 (Fig. 2A). In the co-culture experiments, five million AML cells (obtained from six HLA-A2 positive and three HLA-A2 negative patients) were co-cultured in 24-well G-rex plates with 100 thousand of NFAT GMCAR WT1TCR, or WT1TCR-T cells for 7 days, without cytokines. The remaining live cells were analyzed by FACS by staining with antibodies specific to antigens CD3, CD33, and EGFR. In addition, we performed detailed immunophenotyping of the TCR-T cells with antibodies specific to antigens CD4, CD8, CD62L, CD45RA, CD27, CD28, and PD1 to assess their memory differentiation pattern during the co-culture. This experiment revealed that NFAT GMCAR WT1TCR-T cells, compared to WT1TCR-T cells, eliminated most AML cells (Fig. 3A). Importantly, HLA-A2 negative AML cells were not eliminated by either type of TCR-T cells, thus further demonstrating the dependency of the killing on the TCR-HLA-A2 interaction. Although no significant differences were observed in the ratios of CD4+ and CD8+ EGFR+ T cells between these two types of TCR T cells or in the percentages of PD1-positive T cells (Fig. 3B), we detected significant differences in their immunophenotypes. The memory differentiation pattern of NFAT GMCAR WT1TCR in comparison to WT1TCR was characterized in the CD4-positive subset by nonsignificant differences in the percentages of stem-cell memory T cells, higher percentages of central memory T cells (*P* < ****), lower percentages of effector memory T cells (*P* < ***), and nonsignificant differences in the percentages of T_EMRA_ cells. A similar comparison of the CD8-positive subset revealed that CD8+NFAT GMCAR WT1TCR contained significantly fewer stem-cell memory T cells (*P* < **), more central memory T cells (*P* < **), and nonsignificant differences in the percentage of effector memory T cells and T_EMRA_ cells (Fig. 3C). The co-cultivation of HLA A2+ AML samples (*n* = 6) was repeated with TCR-T cells prepared from two donors; all statistical analyses were performed via paired *t*-tests.

In summary, NFAT GMCAR WT1TCR-T cells have more potent antileukemic activity than WT1TCR against primary AML cells, which is associated with their enhanced expansion and increased percentage of T cells with less differentiated memory immunophenotype mainly among CD4-positive T cells.

### CD3 x CD33 BiTE enhances the antileukemic activity of NFAT GMCAR WT1TCR T cells via the recruitment of nontransfected T cells

**Figure 4.**
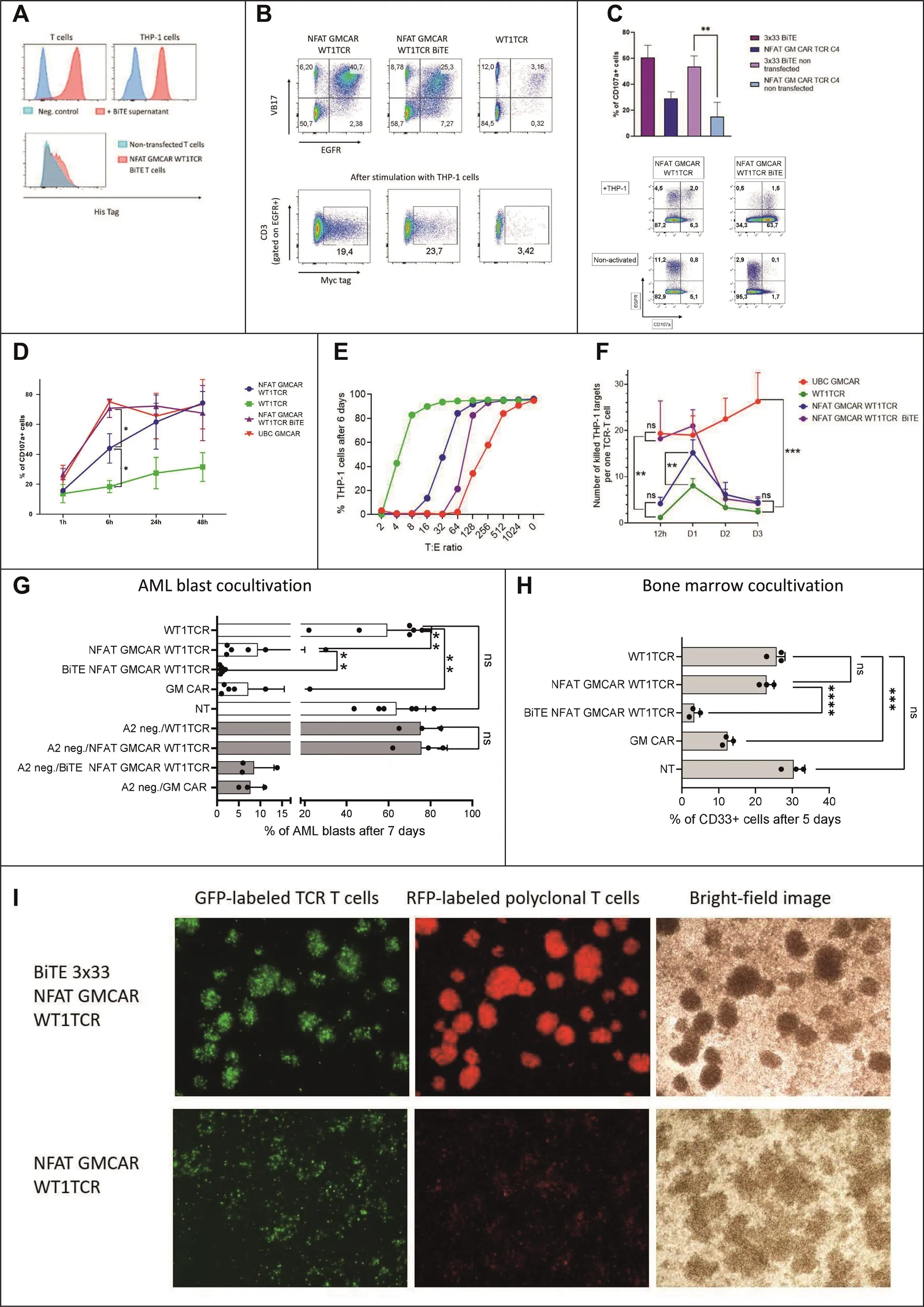
CD3xCD33 BiTE is efficiently expressed by single-vector transposon and enhances the antileukemic function of engineered T cells. (A) The expression of the CD3 x CD33 BiTE in HEK293T cells and NFAT GMCAR WT1TCR BiTE T cells. (B) The expression of individual transgenes by NFAT GMCAR WT1TCR, NFAT GMCAR WT1TCR BiTE, and WT1TCR T cells. (C) The degranulation assay was used to determine whether the BiTE can activate the nontransfected bystander T cells. *t*-test, *n* = 3 (C). The kinetic of this degranulation (D) was assessed by measurement at four time points (1, 6, 24, and 48 hours) using all four studied constructs. *t*-test, *n* = 3. (E) The BiTE induces effective cytotoxicity of engineered T cells in long-term co-cultivation. (F) The BiTE increases the elimination of significantly more THP-1 targets per one TCR-T cell compared to BiTE-negative WT1TCR and NFAT GMCAR WT1TCR T cells. One-way ANOVA,Tukey HSD test (*n* = 3). (G) The BiTE enhances cytotoxicity against primary AML cells. Two-sided paired *t*-test (*n* = 3). (H) NFAT GMCAR WT1TCR BiTE T cells eliminate natural CD33-positive myeloid progenitor cells. Two-sided paired *t*-test, *n* = 3. (I) The effects of CD3x33 BiTE on the forming of activated clusters including both TCR-T and nontransfected T cells.

In the next step, we inserted into the NFAT GMCAR WT1TCR transposon a third expression cassette driven by human cytomegalovirus (CMV) immediate-early enhancer and promoter, which produces a CD3 x CD33 bispecific T-cell engager antibody (BiTE). The CD3 x CD33 BiTE is derived from the AMG330 construct [13]; thus, it is composed of the same scFv segments and contains a His Tag at the C-terminus. First, the expression of the BiTE was verified by transfection of the transposon plasmid into the HEK293 cells. Forty-eight hours post-transfection, the supernatant was collected and used to label THP-1 cells (CD33 positive) and normal T cells, followed by staining with anti-His tag antibody. The histograms in Fig.4A show that THP-1 cells and T cells indeed stained positively with anti-His Tag Ab, while the cells incubated with control supernatant from NT HEK293 cells were negative. Thus, the BiTE is effectively produced by the transposon construct. Next, the same construct (NFAT GMCAR WT1TCR BiTE) was transfected into T cells to evaluate the BiTE expression in T cells. The histograms in Fig.4A (lower panel) show that the NFAT GMCAR WT1TCR BiTE T cells stained less intensively than T cells labeled with the HEK293 supernatant, suggesting a low-level expression of the BiTE by transfected T cells. Next, we determined the expression of the GMCAR, WT1TCRbeta chain, and tEGFR by the NFAT GMCAR WT1TCR BiTE construct. The transfected T cells were detected with anti-TCR Vβ17 and anti-EGFR antibodies; the expression of the NFAT GMCAR on CD3+ EGFR+ T cells was detected with anti-Myc tag Ab after stimulation with THP-1 cells for 24 hours. The FACS dot plots (Fig. 4B, upper panel) indicate that the expression of the WT1TCRbeta chain by the BiTE construct was partially reduced compared to NFAT GMCAR WT1TCR and WT1TCR. Additionally, we detected similar expression of the tEGFR and GMCAR between NFAT GMCAR WT1TCR and NFAT GMCAR WT1TCR BiTE constructs (Fig. 4B, lower panel). To determine the biological activity of the BiTE, T cells were transfected with NFAT GMCAR WT1TCR or NFAT GMCAR WT1TCR BiTE transposons and co-cultivated with THP-1 targets (Fig. 4C and D) to determine the effects of the BiTE on the degranulation of nonmodified T cells (EGFR negative). NFAT GMCAR WT1TCR and NFAT GMCAR WT1TCR BiTE T cells were mixed with THP-1 targets (E:T = 1:3), and the degranulation was detected with CD107a antibody after 6 hours. In this assay, the NFAT GMCAR WT1TCR BiTE T cells demonstrated stronger degranulation compared to NFAT GMCAR WT1TCR T cells (mean 60, 6% vs. 29% out of EGFR+ CD3+ cells). Moreover, the BiTE induced substantial degranulation of the NT (i.e. EGFR negative) CD3+ T cells (mean 53, 6% vs. 15%). The degranulation kinetics revealed a rapid cytotoxicity onset among BiTE-expressing T cells similar to UBC GMCAR T cells (Fig. 4D).

The ability of CD3x33 BiTE to recruit nonmodified T cells for target elimination was visualized by fluorescent microscopy using GFP-labelled TCR-T cells (NFAT GMCAR WT1 TCR, or NFAT GMCAR WT1 TCR BiTE) and T cells modified only with transposon expressing RFP (polyclonal T cells) (Fig. 4I). GFP+ and RFP+ T cells were mixed with THP-1 targets, co-cultivated for 2 days and then visualized by fluorescent microscopy. Secreted BiTE effectively recruited polyclonal RFP+ T cells toward the GFP+ TCR-T cell clusters formed to eliminate the targets.

To further characterize the kinetics of killing of the target cells by NFAT GMCAR WT1TCR BiTE T cells, we used a caspase-3/7 fluorescent substrate to calculate the number of killed targets per one TCR-T cell (Fig. 4F). TCR-T cells were co-cultured at initial E:T = 1:50, and at defined time points, the samples were stained with the caspase-3/7 substrate in combination with anti-CD3 and anti-EGFR antibodies and DAPI dye. This experiment revealed that the BiTE-expressing T cells had very strong initial cytotoxicity comparable to UBC GMCAR (average 20 killed targets per T cell at 12-hour time point), which was significantly more robust than the cytotoxicity of both NFAT GMCAR WT1TCR and WT1TCR T cells (*P* < **). However, in the next time points (Day 1, Day 2, and Day 3), the cytotoxicity of the UBC GMCAR persisted at similar levels, but the cytotoxicity of WT1TCR, NFAT GMCAR WT1 TCR, and NFAT GMCAR WT1TCR BiTE T cells peaked at Day 1 time point and then gradually decreased. However, in agreement with our previous experiments, the cytotoxicity of the WT1TCR T cells was significantly lower at the peak of the response (Day 1) compared to NFAT GMCAR WT1 TCR T cells (8 vs. 17 killed targets/T cell, average value, *P* < *) and NFAT GMCAR WT1TCR BiTE T cells (8 vs. 24 killed targets/T cell, average value, *P* < *, *n* = 3).

We additionally compared the cytotoxic strength of the described types of T cells by titrating the E:T ratio to identify the limiting number of T cells required to eliminate all THP-1 targets during a 6-day co-culture assay (Fig. 4E). In agreement with our previous experiments, we observed that the most potent killers were UBC GMCAR T cells—they killed all THP-1 targets at the highest E:T ratio (1:64–128). The comparison of the three types of TCR-T cells (WT1TCR, NFAT GMCAR WT1 TCR, NFAT GMCAR WT1TCR BiTE) revealed that the BiTE T cells were the most potent effectors because they eliminated all THP-1 targets at a 1:32–64 ratio compared to a 1:8–16 ratio of NFAT GMCAR WT1 TCR and a 1:2–4 ratio of WT1TCR T cells.

### NFAT GMCAR WT1TCR BiTE T cells have strong antileukemic activity against primary AML cells but at the price of CD33-specific myelotoxicity

Next, analogically to the experiment presented in Fig. 3, we tested the BiTE T cells against primary AML cells using the same set of AML samples. Similarly, we observed an efficient elimination of the AML cells by NFAT GMCAR WT1TCR T cells. The engineered T cells were cultivated with primary AML cells (*n* = 6) at E:T = 1:50 ratio; after 7 days, the percentages of live AML targets and effector EGFR+ T cells were measured by FACS with antibodies specific to the antigens CD123, CD3, and EGFR. The control groups included NT T cells and HLA-A2 negative AML cells. On average, 6.5% of AML cells were detected out of all live cells after 7 days (*n* = 6), while in the NT T-cell group, an average of 66% of AML cells were detected. Interestingly, the BiTE T cells showed more robust effects not only against the HLA A2 positive AML cells (in average 1.2% of remaining AML cells, *P* < *), but importantly, they also partially eliminated the HLA A2 negative cells, suggesting that the BiTE effectively induces killing of targets independently of the HLA A2 (Fig. 4G and H). Next, we carried out BM cell co-culture assays to evaluate the potential hematological toxicity of the described TCR-T cells (especially against myeloid-derived progenitor cells) (Fig. 4H). First, the BM cells (HLA-A2 negative) were expanded in a complete SFMII media for 5 days, then were mixed (5 million cells per well) with THP-1 cells and the described TCR-T cells (each 100 thousand per well). After 3 days of cultivation, the remaining populations of BM-derived cells were detected by FACS using antibodies to HLA-A2, CD33, CD14, CD3, and EGFR. The presented results (one representative experiment out of three) indicate that the BiTE T cells eliminated the majority of CD33-positive cells, including THP-1 and CD33+CD14+ monocytic lineage cells, which express high levels of CD116 (GM-CSF receptor). In contrast, both the WT1TCR T cells and NFAT GMCAR WT1TCR T cells were not myelotoxic.

In summary, the BiTE redirects T cells against the CD33 antigen, resulting in effective antileukemic activity independent of HLA-A2.

### Analysis of the T-cell Signaling network by phospho-flow reveals stronger activation pathways induced by NFAT GMCAR

**Figure 5.**
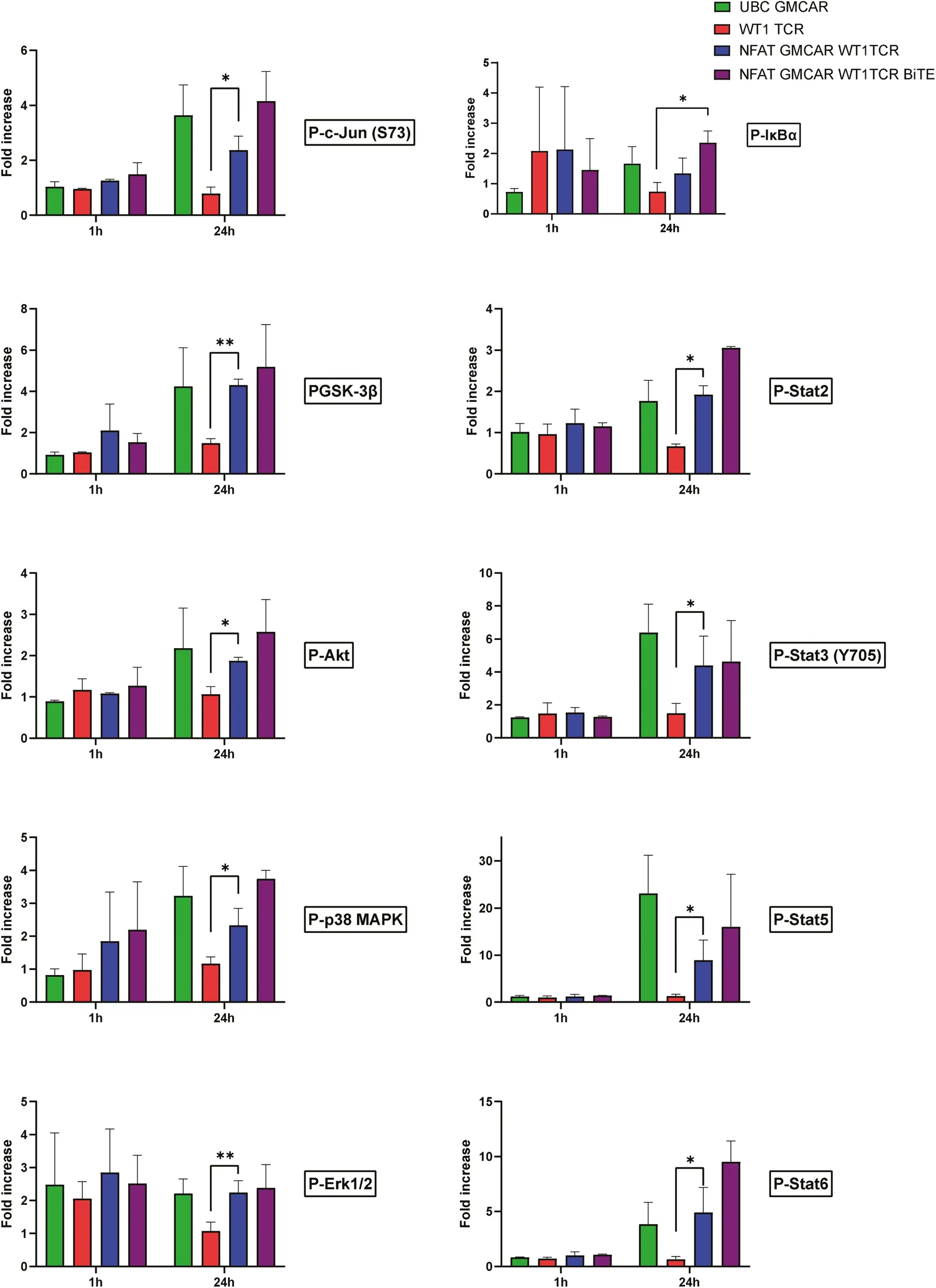
NFAT-inducible GM CAR increases activation of key signaling phosphorylases after antigenic stimulation by WT1 TCR.

Intracellular flow cytometry using phospho-site specific antibodies (phospho-flow) is a powerful technology for studying phosphorylation signatures in primary T cells after antigen-specific activation. We have measured the kinetics and the activatory status of several key kinases (c-Jun, PGSK-3ß, Akt, p38 MAPK, Erk ½, IkBa, Stat2, 3, 5, 6) in all four types of studied T cells during co-cultivation with THP-1 targets (Fig. 5 and Supplementary Fig. S3). The cells were mixed at an E:T ratio of 1:5; after 1 and 24 hours, the cells were analyzed by phospho-flow to determine the activation status of the aforementioned signaling kinases. We have observed a significant positive effect of the NFAT-inducible GMCAR transgene on the activation status of these indicated pathways in comparison to WT1TCR T cells after 24 hours. The graphs present a ratio of MFI in activated T cells divided by MFI in nonactivated T cells at each time point to show a fold increase in the kinase phosphorylation intensity (*t*-test, *n* = 3). These results further demonstrate the positive effects of the NFAT GMCAR on promoting WT1-triggered T-cell activation.

## Discussion

**Figure 6.**
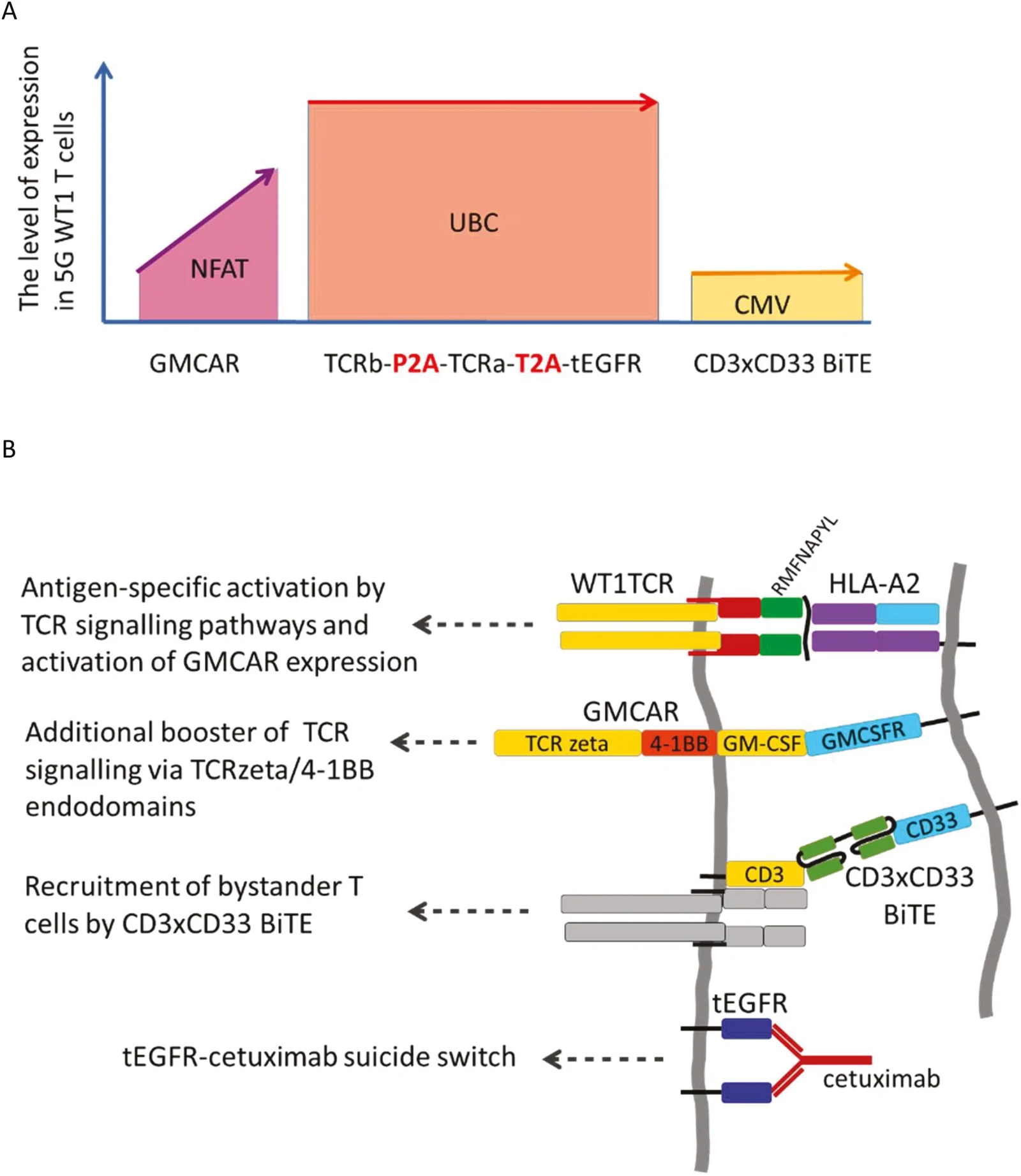
The scheme of the properties of the 5G WT1 TCR T cells. (A) T cells engineered with the 5G transposon construct (i.e. NFAT GMCAR WT1TCR BiTE) express five individual proteins from three promoters, all oriented in the 5’–3’ direction. Upon integration into the genome, the NFAT-regulated promoter composed of six NFAT-binding DNA domains followed by minimal IL2 promoter drives the transcription of the GMCAR transgene upon antigenic stimulation. The second transgene is constitutively expressed as a single transcript under a strong ubiquitin (UBC) promoter. It comprises three proteins—TCR beta, TCR alpha, and truncated EGFR receptor, separated by P2A and T2A peptides, respectively, which induce ribosomal skipping during the translation. The third transgene is the CD3 x CD33 BiTE driven by the minimal CMV promoter, which produces low-level expression in T cells. (B) The scheme summarizes the individual transgenes’ combined effects on engineered T cells’ functionality. Four specific properties were introduced into T cells via the 5G transposons: Firstly, WT1TCR composed of alpha and beta chains leads to the recognition of RMFNAPYL/HLA-A2 antigenic complex and a subsequent activation mimicking the natural stimulation of T cells via the endogenous TCR. Secondly, the inducible expression of the GMCAR recognizing GM-CSF receptor enhances WT1-specific activation but is too low to initiate the cytotoxicity directly. Thirdly, the secreted CD3xCD33 bispecific T-cell engager (BiTE) targets widely expressed myeloid antigen CD33 not only by the transfected T cells but also by the nontransfected T cells and increases the overall killing capacity but at the price of CD33-dependent myelotoxicity. Finally, the inserted truncated EGFR-based receptor enables the specific elimination of transfected T cells in vivo via the pharmaceutical-grade antibody cetuximab.

Cancer T-cell immunotherapy with TCR-engineered T cell is an attractive approach for specific targeting of tumor cells. TCR-T cells are currently evaluated in numerous clinical trials; however, the HLA-encoding genes are the most polymorphic in the human genome, with over 20 000 HLA-class I alleles identified to date [18]. For this reason, TCR-T therapies typically utilize TCRs restricted to relatively common HLA alleles and antigens, such as HLA-A*02:01 [19] which enables a feasible TCR optimization for effective tumor engagement. Herein, we focused on an alternative approach to enhance TCR functions—instead of modifying the TCR sequence for enhanced expression and functions, we engineered T cells with a single-vector construct to express three molecular ‘guns’—CAR, TCR, and BiTE, and a tEGFR receptor as a cell suicide system (Fig. 6). Our results demonstrate that the WT1TCR specific to the RMFNAPYL/HLA-A2 complex functioned as a primary activatory signal. At the same time, the inducible GMCAR construct enhanced T-cell activation upon WT1TCR engagement, but it could not trigger cytotoxicity toward its ligand—the GM-CSF receptor. Upon WT1TCR activation, GMCAR expression increases, providing a booster for T-cell activation via recognition of its target, the GM-CSF receptor, resulting in enhanced proliferation and maintenance of strong cytotoxicity in NFAT GMCAR WT1TCR T cells. When stimulated and stained for several phospho-kinases, we have shown that the WT1TCR and NFAT GMCAR WR1TCR T cells have significantly different activation profiles. This further supports the hypothesis that the addition of GMCAR under an inducible promoter enhances the activation intensity of modified T cells, thereby improving their activity and expansion during the recognition of tumor targets. Importantly, HLA A2 negative/GM-CSF receptor positive targets (K562 cell line) or primary AML cells from HLA A2 negative patients were not efficiently killed by NFAT GMCAR WT1TCR T cells, indicating that the NFAT-driven expression of the GMCAR is too low to initiate potent cytotoxicity comparable to T cells expressing constitutive UBC GMCAR transgene. Most importantly, the sensitivity of NFAT GMCAR to pharmacological inhibition via cyclosporine provides an additional level of safety in addition to the EGFR safety switch and thus supports a possible clinical application. The presented mechanism of enhancement of the WT1TCR signaling by NFAT-regulated CAR can be modified to target different WT1-positive tumors, such as ovarian and lung carcinoma, by using inducible CAR specific to surface antigens such as mesothelin, Her2, or folate receptor alpha.

Next, the insertion of the CD3 x CD33 BiTE (which is derived from AMG330 antibody constructs [20]) into the transposon equipped the engineered T cells with different and interesting antitumor functions. A similar approach has been described by Choi *et al.* in a glioblastoma mouse model using CAR-T + BiTE cells secreting EGFR-specific BiTEs that redirected CAR-T cells and recruited nontransduced bystander T cells against wild-type EGFR [21]. In general, the variability in clinical characteristics of various malignant tumors poses a challenge for cellular immunotherapy. For example, one goal could be the effective removal of large bulky tumors and the long-term persistence of administered T cells to prevent a relapse in patients with B-cell lymphomas treated with CD19 CAR-T therapy. A different goal might focus on a consolidation treatment after intensive standard therapy to remove residual leukemia burden before planned HSCT, for example, in patients with AML.

On the other hand, targeting of common myeloid antigens (such as CD33, CD133, CLEC12A) by long-term persisting CAR T-cells could lead to severe off-target toxicity such as prolonged cytopenias [22–26]. Therefore, it might be beneficial to develop T-cells with strong antileukemic effects specifically localized into the tumor stroma but with reduced long-term persistence and complemented with a suicide gene system. For this reason, we selected a CMV promoter to drive the expression of the CD3xCD33 BiTE because the CMV promoter becomes partially inhibited in primary human T cells [27]. Besides this, we added the tEGFR molecule to the expression cassette to enable selective depletion of the modified T cells. Under these settings, the WT1TCR engagement enhances local CD3xCD33 BiTE production by expanding and maintaining the very TCR-T cell population that secretes BiTEs. This could possibly be useful in the case of MRD where remaining NFAT GMCAR WT1TCR BiTE cells would reactivate upon contact with WT1-positive tumor cells, proliferate, and eliminate the malignant remnants. This means that the source of the BiTE in this case is self-renewable and can also be abolished upon demand.

Although we have observed the effective elimination of CD33+ cells in BM co-culture experiments in vitro (Fig. 4H), the short-lived cytotoxicity of NFAT GMCAR WT1TCR T cells compared to UBC GM CAR T cells could reduce the risk of generalized myeloablation. Furthermore, the localized targeting of CD33+ cells by the BiTE in the BM might prevent the selection of HLA-loss AML clones; moreover, the CD33 antigen is also expressed on myeloid-derived suppressor cells, which are involved in hampering immune-based therapies [28]. However, the myelotoxicity that inevitably follows the use of CD3xCD33 BiTE is a nondismissible complication that needs to be considered when implementing such treatment.

Although the high-avidity TCRs against common tumor antigens can be generated in advance, this task is challenging for other less frequent HLA alleles. For example, the WT1-specific TCR used in our study was prepared by screening tens of healthy donors [7] and engineered to reduce cross-pairing with endogenous TCRs—such an approach is not possible for patients with rapidly progressing cancers with limited therapeutic intervention time. The TCR-based cancer therapy in these patients requires a rapid method for producing TCR-T cells that simultaneously does not require thorough safety testing. For example, therapy with MART-1 and MAGE-A3-specific TCR in melanoma patients [29–31] produced fatal cardiotoxicity because of the high expression of MART-1 and MAGE-A3 in heart tissue. Similarly, Parkhurst *et al*. found severe colitis in three patients with metastatic colorectal cancer treated with TCR-T cells targeting CEA (691–699) [32]. These known issues of both on-target off-tumor and off-target toxicities support our described strategy of combining a relatively weak tumor-specific TCR and an inducible CAR. This strategy might effectively enhance the antitumor effects of nonengineered (and thus safer) patient-derived autologous TCRs, which can be isolated directly from cancer patients.

The most commonly used method of CAR-T cell manufacturing is viral transduction. Compared to the piggyBac transposon system, the production and quality control of the CAR-T cells is relatively slow. The packaging capacity of transposon vectors is higher than that of viral vectors, albeit not unlimited. We have shown that larger constructs may lead to lower modification efficacy. This can be compensated by using a higher amount of DNA per electroporation—the cells should be modified by DNA amount containing a normalized number of transgene copies. Additionally, GMP-grade viral vectors are considerably more expensive, and with the financial burden that comes with the necessary establishment of the GMP facility, the piggyBac comes out as a better option for noncommercial subjects [33].

Aside from the earlier established use of viral vectors, modern complex T-cell gene engineering technologies primarily rely on CRISPR/Cas9 DNA editing, enabling locus-specific integration of large inserts. However, there is an indication that this method might produce unwanted genotoxicity, such as megabase-large DNA deletions, chromosomal translocations, and chromothripsis [34–38]. Similarly, the genotoxicity of piggyBac transposon was also reported [39, 40]. The CRISPR/Cas9 method enables the ablation of endogenous TCR. However, this can be substituted by other approaches such as protein expression blockers (PEBLs) [41], which can be combined with piggyBac transposons.

Our approach relies on the electroporation of a single plasmid vector (the transposase can be delivered by mRNA) and is technically straightforward, rapid, and cost-effective. Furthermore, a single-vector modification could minimize the risk of insertional mutagenesis and improve the likelihood of approval by major regulatory bodies for clinical translation compared to strategies relying on the insertion of multiple transgenes. Importantly, our transposon vector contains an EGFR-based suicide system that enables the targeted elimination of engineered T cells by the therapeutic antibody cetuximab. This feature serves not only as a safety precaution but can be used to eliminate 5G WT1 TCR-T cells at a defined time point upon administration to patients.

During our experiments, we performed functional tests to elucidate how the combination of transgenic TCR, CAR, and a BiTE affects the activation capacity, intracellular signaling, and phenotype of modified T cells and how the combination of said molecules influences their ability to kill target cells. To mimic a physiological environment *in vitro*, we used a BM-derived culture using samples from AML-diagnosed patients. To further characterize the modified T cells, experiments including an *in vivo* model might provide additional data, for example, influence of the tumor microenvironment or possible on-target off-cancer toxicity; however, current murine models of AML have several limitations and might provide only partial answers [42].

In summary, herein, we present a novel strategy to enhance antitumor potency and the safety of T cells through complex redirection of T-cell specificity via insertion of TCR, CAR, and BiTE constructs and a tEGFR gene suicide system. The presented expression platform enables complex nonviral transgenesis of T cells via a single vector. These successful data support further development and subsequent clinical translation of 5G TCR-T cells for the treatment of AML.

## Material and Methods

### Cell source

Peripheral blood mononuclear cells (PBMCs) were isolated from buffy coats obtained from blood donors. AML cells were obtained from newly diagnosed patients (*n* = 8) having a high blast cell count in the peripheral blood and, therefore, were indicated for a leukodepletion before initiation of the chemotherapy. The expression of WT1 and HLA-A2 was determined in our Institution as part of standard hematological examinations of patients with AML. BM aspirates and matched samples of PBMCs were obtained from patients undergoing trepanobiopsies as part of complex hematological examinations. These patients had mild cytopenia (max. Grade 1), no detectable blasts in the BM, and later were not diagnosed with any hematological disease. Isolated AML blasts, peripheral blood samples, and BM aspirates were purified by Ficoll-Paque gradient centrifugation, cryopreserved, and stored in liquid nitrogen. The cell lines THP-1, K562, and Ramos were obtained from DSMZ, Germany.

### DNA plasmids and sequences

All sequences were prepared by gene synthesis and assembled by standard restriction endonuclease-based cloning techniques into parental piggyBac (PB) transposon vector containing UBC promoter [16]. The UBC promoter design is similar to the published report [10]. The transposase-expressing vector contains the hyperactive piggyBac transposase driven by CMV promoter [43]. The amino acid sequences of individual constructs are provided in the Supplementary Material. Plasmids were purified by standard techniques using Plasmid Midi/Maxi kits (Qiagen, Germany).

### Preparation of gene-engineered T cells and their cultivation

For all experiments, we used CellGenix GMP DC medium (CellGenix, Germany) supplemented with 10% heat-inactivated 10% FCS (Gibco, USA) and antibiotics penicillin and streptomycin (Gibco, USA).

The PBMCs were prepared from buffy coats by gradient centrifugation using Ficoll-Paque Premium (GE Healthcare) after separation cells were transfected with the Neon electroporator (Thermo Fischer Scientific, USA) similarly as described previously [16, 44]. Briefly, 1 × 10e7 cells were resuspended in 100 μl buffer T containing 2 μg of transposon plasmid and 1 μg of transposase plasmid and were electroporated (ELP) in 100 μl tips by one pulse 20 ms/2300 V. To generate the “BiTE” T cell, the amount of transposon plasmid was increased to 4 μg per ELP.

To generate fluorescently labeled TCR-T cells, PBMCs were electroporated with plasmids containing additionally 1 μg of GFR/RFP expressing piggyBac transposons plasmids per 100 μl reaction.

Cells were cultured in cell media with cytokines IL-7 and IL-15 (10 ng/ml each, Peprotech, UK) and activated with TransAct (Miltenyi Biotec, Germany) 1 day after ELP. At Day 7, cells were restimulated with plate-bound TCR Vβ17 antibody.

### Antibodies and FACS

The following antibodies were used to detect the expression of the transgenes: Alexa Fluor® 647-labeled anti-EGFR Ab (clone # 423103, Bio-Techne R&D Systems, USA), Alexa Fluor® 647-labeled anti-His tag Ab (Mouse IgG1, clone # AD1.1.10, Bio-Techne R&D Systems, USA) and anti-Myc tag-FITC (clone # SH1-26E7.1.3, Miltenyi Biotec, USA). The WT1TCR expression was visualized with APC-labeled anti-Vβ17 Ab (clone # REA915, Miltenyi Biotec, Germany). To determine the immunophenotype of T cells, the following antibodies were used: mouse anti-human CD3-BV786 (clone # UCHT1), mouse anti-human CD45RA-BUV737 (clone # HI100), mouse anti-human CD62L-BV650 (clone # DREG-56), mouse anti-human PD1-BB700 (clone # EH12.1), mouse anti-human CD4-Pacific Blue (clone # RPA-T4), mouse anti-human CD69-BV786 (clone FN50), all from BD Biosciences, USA. Mouse anti-human CD27-APC-Cy7 (clone # LT27), mouse anti-human CD28-PE-Cy7 (clone # CD28.2), mouse anti-human CD8-AF700 (clone # MEM-31), and mouse anti-human HLA-A2-Alexa Fluor® 488 (mouse monoclonal IgG2b, clone # BB7.2) and mouse anti-human CD107a-APC (clone # H4A3), were from Exbio, Czech Republic (CZ). The cells were stained with antibodies in FACS staining buffer (PBS % 1% FCS and 0.1% sodium azide) for 30 minutes at 4°C.

Live cells were identified using a LIVE/DEAD stain kit (Thermofisher Scientific, USA) or with DAPI, depending on the assay. FACS samples were analyzed with the BD Fortessa instrument, FACS data were processed by FlowJo software, and statistical analysis was performed with GraphPad Prism software using indicated statistical tests.

### *In vitro* assays

The cytotoxic tests were performed using indicated AML cell lines (THP-1, K562) labeled with Tag-it-violet or Carboxyfluorescein succinimidyl ester (CFSE) dyes (Thermofisher Scientific, USA) according to manufacturer’s instructions and were cultivated with engineered T cells at indicated ratios. The co-cultivation assays with primary AML cells were performed with thawed AML samples by mixing 5 million AML cells + 100 thousand effector TCR-T cells (E:T ratio = 1:50) in 24-well G-rex plates (Wilson Wolf, USA) in CellGenix media without cytokines. On Day 3, cells were fed with fresh media. The surviving subsets were analyzed on Days 3 and 7 by FACS in a staining buffer supplemented with 1% human IgG to block Fc receptors present on myeloid AML cells. The percentages of dead/live target cells were determined by FACS with indicated antibodies and DAPI dye.

Peptide stimulations were performed using TCR-T cells from HLA-A2 positive donors. TCR-T cells were washed from cytokines and rested for 48 hours in media; then, peptides at indicated concentrations were added to cells. The peptides WT1/RMFNAPYL and CMV pp65/NLVPMVATV were obtained from JPT Peptide Technologies GmbH, Germany. CMV-specific T cells were identified with hamster anti-mouse TCRβ chain-APC antibody (clone # H57-597, BioLegend, USA). The activation was measured with antibodies to antigens CD69, CD3, EGFR, and CD8 after 24 hours.

To visualize the cytotoxicity of TCR-T cells by microscopy, the THP-1 targets were mixed with effector T cells labeled with GFP/RFP transposons and were cultivated for up to 14 days in independent wells. The cells were analyzed by FACS to determine the numbers of GFP/RFP+EGFR+ TCR-T cells at indicated time points. On Day 7, the images of clusters of THP-1 targets with effector T cells in the cultivation wells were observed at ×20 magnification on an Olympus IX70 microscope with GFP/RFP filter cubes.

To determine the degranulation of TCR-T cells, an APC-labelled CD107a antibody was added to cells at 1:100 dilution, and cells were further incubated for 4 hours at 37°C; then, the cells were washed and labeled with anti-CD3 Ab, anti-CD69 Ab, and anti-EGFR Ab. TCR-T cells cultured without target cells were used as a negative control.

To calculate the number of killed targets per TCR-T cell, 5 million THP-1 cells were mixed with 100 thousand TCR-T cells or UBC GMCAR T cells in 5 mil media (E:T = 1:50) in 6-well Gas-permeable Culture Plates (Miltenyi Biotec, Germany). At each time point, the samples were labeled with a caspase-3/7 green fluorescent substrate (CellEvent, Thermofisher Scientific, USA) at 1:1000 dilution in combination with antibodies to EGFR and CD3 by incubation for 30 minutes at room temperature in cell media. The cells were then centrifuged (600 g—5 minutes), resuspended in PBS containing DAPI dye, and analyzed by FACS. The killing capacity was calculated as the number of caspase+CD3-DAPI− cells divided by the number of CD3+ EGFR+ cells per sample. An example of the gating strategy is presented in Supplementary Fig. 1.

The BM cell assay was done in complete StemSpan SFMII media containing 10% FCS and supplemented with StemSpan CD34+ Expansion Supplement containing Flt3L, SCF, IL-3, IL-6, and thrombopoietin (StemCell Technologies, USA). The BM cells (HLA-A2 negative) were thawed and expanded in complete SFMII media for 5 days before mixing with T cells. The TCR-T cells were prepared from PBMCs of the same donors without restimulation with TCR VB17 Ab and were rested in media without cytokines for 1 day before co-cultivation. The co-cultivation assay mimicking human hematopoiesis infiltrated with AML cells was carried out in 24-well G-rex plates with the initial number of 5 million BM cells, 50 000 TCR-T cells, and 50 000 THP-1 cells per well. After 3 days, the percentages of CD33+CD14+ myelomonocytic lineage cells were determined by FACS using antibodies to CD33, CD14, CD3, EGFR, and HLA-A2.

### Phopho-flow

The protocol was adopted from Cell Signaling Technology. Briefly, cells were washed with cold PBS and stained for extracellular markers (EGFR-AF647 [R&D Biosystems FAB9577R] or Myc-FITC [Miltenyi Biotec 130-116-485]) for 10 minutes on ice. The cells were rewashed with PBS and were fixated in 4% formaldehyde (100 µl per 1e6 cells) for 15 minutes at RT, followed by another wash, after which the samples were resuspended in PBS and permeabilized by slowly adding ice-cold 100% methanol to a final concentration of 90% while gently vortexing. The samples were incubated for 10 minutes on ice or overnight at −20°C. The methanol was washed away with PBS, and the cells were aliquoted to a 96-well plate. Staining for phospho-antigens with primary Rabbit antibodies (1:100 dilution in FACS buffer) for 1 hour at RT. The plate was 2× washed with PBS, and samples were stained by secondary antibody Goat anti-Rabbit Ig-PE (1:200 dilution in FACS buffer) for 30 minutes at RT. After another 2× wash with PBS, the samples were resuspended in PBS and analyzed on a flow cytometer.

### Statistical analysis

Statistical analysis was performed using GraphPad Prism software (Version 8.4.2), and consultations were provided by bioinformatician Ing. Pavla Pecherková, PhD (Institute of Hematology and Blood Transfusion). Respective statistical tests used are indicated in figure legends. Samples were paired when appropriate. Error bars show SD. Indicated significance levels used are *P* <.05 *, *P* <.01 **, *P* <.001 ***, *P* <.0001 ****.

## Supplementary Material

ltaf022_suppl_Supplementary_Figures_S1-S3

## Data Availability

The data underlying this article are available in the article and in its Supplementary Material. Further supplementary data underlying this article will be shared on reasonable request to the corresponding author.
