# Correction to: Targeting of acute myeloid leukemia by five-gene engineered T cells expressing transgenic T-cell receptor specific to WT1, chimeric antigenic receptor specific to GM-CSF receptor, bispecific T-cell engager specific to CD33, and tEGFR suicide gene system

**DOI:** 10.1093/immadv/ltag025

**Published:** 2026-10-30

This is a correction to: Kristýna Šmilauerová, Martin Štach, Martin Mucha, Šárka Vaníková, Jana Rychlá, Pavel Otáhal, Targeting of acute myeloid leukemia by five-gene engineered T cells expressing transgenic T-cell receptor specific to WT1, chimeric antigenic receptor specific to GM-CSF receptor, bispecific T-cell engager specific to CD33, and tEGFR suicide gene system, *Immunotherapy Advances*, Volume 5, Issue 1, 2025, ltaf022, https://doi.org/10.1093/immadv/ltaf022

In the originally published version of this manuscript, the colours for the red and green graph lines in Figure 4E were reversed. The correct colours have now been reinstated.

This error has been corrected.

