# Supplementary material for: Targeting of acute myeloid leukemia by five-gene engineered T cells expressing transgenic T-cell receptor specific to WT1, chimeric antigenic receptor specific to GM-CSF receptor, bispecific T-cell engager specific to CD33, and tEGFR suicide gene system
Source: Immunother Adv. 2025 Jun 11;5(1):ltaf022. doi: 10.1093/immadv/ltaf022 (PMC12306182; doi:10.1093/immadv/ltaf022)
Supplement: ltaf022_suppl_Supplementary_Figures_S1-S3 [file ltaf022_suppl_supplementary_figures_s1-s3.docx]

**Figure S1**

|  |
| --- |
| **Fig. S1** **WT1TCR T cell cocultivation with THP-1 tumour cell line.** WT1TCR T cells were cocultured with THP-1 cells at initial E:T = 1:50 for up to three days during which the intensity of killing was measured with caspase 3/7 green fluorescent substrate and with CD3 and EGFR antibodies to calculate the number of freshly killed targets (identified as DAPI negative, CAS3/7 positive) per one TCR-T. The image shows an example of the FACS detection method. |

**Figure S2**

| **GMCAR**  MWLQSLLLLGTVACSISAPARSPSPSTQPWEHVNAIQEARRLLNLSRDTAAEMNETVEVISEMFDLQEPTCLQTRLELYKQGLRGSLTKLKGPLTMMASHYKQHCPPTPETSCATQIITFESFKENLKDFLLVIPFDCWEPVQEGEQKLISEEDLGLLGPYELWELSHPTTTPAPRPPTPAPTIASQPLSLRPEACRPAAGGAVHTRGLDFACDIYIWAPLAGTCGVLLLSLVITLYCNHRNRRRVKRGRKKLLYIFKQPFMRPVQTTQEEDGCSCRFPEEEEGGCELRVKFSRSADAPAYQQGQNQLYNELNLGRREEYDVLDKRRGRDPEMGGKPQRRKNPQEGLYNELQKDKMAEAYSEIGMKGERRRGKGHDGLYQGLSTATKDTYDALHMQALPPR  Blue – GM CSF, green – myc tag, orange – hinge, grey – transmembrane region, magenta – 4-1 BB, ochre – signalling domain. |
| --- |
| **WT1TCR/tEGFR**  MSNQVLCCVVLCFLGANTVDGGITQSPKYLFRKEGQNVTLSCEQNLNHDAMYWYRQDPGQGLRLIYYSQIVNDFQKGDIAEGYSVSREKKESFPLTVTSAQKNPTAFYLCASSPGALYEQYFGPGTRLTVTEDLKNVFPPEVAVFEPSEAEISHTQKATLVCLATGFYPDHVELSWWVNGKEVHSGVCTDPQPLKEQPALNDSRYCLSSRLRVSATFWQNPRNHFRCQVQFYGLSENDEWTQDRAKPVTQIVSAEAWGRADCGFTSESYQQGVLSATILYEILLGKATLYAVLVSALVLMAMVKRKDSRGGSGATNFSLLKQAGDVEENPGPMTSIRAVFIFLWLQLDLVNGENVEQHPSTLSVQEGDSAVIKCTYSDSASNYFPWYKQELGKRPQLIIDIRSNVGEKKDQRIAVTLNKTAKHFSLHITETQPEDSAVYFCAATEDLTLIWGAGTKLIIKPDIQNPDPAVYQLRDSKSSDKSVCLFTDFDSQTNVSQSKDSDVYITDKCVLDMRSMDFKSNSAVAWSNKSDFACANAFNNSIIPEDTFFPSPESSCDVKLVEKSFETDTNLNFQNLSVIGFRILLLKVAGFNLLMTLRLWSSEGRGSLLTCGDVEENPGPMLLLVTSLLLCELPHPAFLLIPCRKVCNGIGIGEFKDSLSINATNIKHFKNCTSISGDLHILPVAFRGDSFTHTPPLDPQELDILKTVKEITGFLLIQAWPENRTDLHAFENLEIIRGRTKQHGQFSLAVVSLNITSLGLRSLKEISDGDVIISGNKNLCYANTINWKKLFGTSGQKTKIISNRGENSCKATGQVCHALCSPEGCWGPEPRDCVSCRNVSRGRECVDKCNLLEGEPREFVENSECIQCHPECLPQAMNITCTGRGPDNCIQCAHYIDGPHCVKTCPAGVMGENNTLVWKYADAGHVCHLCHPNCTYGCTGPGLEGCPTNGPKIPSIATGMVGALLLLLVVALGIGLFMRRRHIVRKR  Blue – TCR beta chain, violet – TCR alpha chain, green – tEGFR, orange – self-cleaving peptides, black – GM CSF signal peptide |
| **BiTE**  MKWVTFISLLFLFSSAYSQVQLVQSGAEVKKPGESVKVSCKASGYTFTNYGMNWVKQAPGQGLEWMGWINTYTGEPTYADKFQGRVTMTTDTSTSTAYMEIRNLGGDDTAVYYCARWSWSDGYYVYFDYWGQGTSVTVSSGGGGSGGGGSGGGGSDIVMTQSPDSLTVSLGERTTINCKSSQSVLDSSTNKNSLAWYQQKPGQPPKLLLSWASTRESGIPDRFSGSGSGTDFTLTIDSPQPEDSATYYCQQSAHFPITFGQGTRLEIKSGGGGSEVQLVESGGGLVQPGGSLKLSCAASGFTFNKYAMNWVRQAPGKGLEWVARIRSKYNNYATYYADSVKDRFTISRDDSKNTAYLQMNNLKTEDTAVYYCVRHGNFGNSYISYWAYWGQGTLVTVSSGGGGSGGGGSGGGGSQTVVTQEPSLTVSPGGTVTLTCGSSTGAVTSGNYPNWVQQKPGQAPRGLIGGTKFLAPGTPARFSGSLLGGKAALTLSGVQPEDEAEYYCVLWYSNRWVFGGGTKLTVLHHHHHH  Green – CD3 IgV heavy, ochre – CD3 IgV light kappa, orange – CD33 IgV heavy, violet – IgV light lambda, blue – linker, black – His tag |
| **Fig. S2** **The sequences of produced TCR-CAR constructs.** The amino acid sequences of GMCAR, WT1TCR/tEGFR, and CD3xCD33 BiTE |

**Figure S3**

| UBC GMCAR WT1TCR NFAT GMCAR NFAT GMCAR  WT1 TCR WT1 TCR BiTE  |
| --- |
| **Fig. S3** **Phospho-flow analysis of TCR-CAR-T cells upon stimulation.** The abundance of phospho-antigens in T cells modified by different TCR-CAR constructs after 24h (orange), and 1h (blue) stimulation with THP-1 cells, and unstimulated CAR-T cells (red). |

**Table S1** **List of used antibodies.** Antibodies by Cell Signalling Technology for the phospho-flow assay.

| Antibody | Cat. No |
| --- | --- |
| Phospho-IκBα (Ser32) (14D4) Rabbit mAb | 2859 |
| Phospho-PTEN (Ser380) Antibody | 9551 |
| Phospho-GSK-3β (Ser9) (D85E12) XP® Rabbit mAb | 5558 |
| Phospho-c-Raf (Ser259) Antibody | 9421 |
| Phospho-Akt (Ser473) (D9E) XP® Rabbit mAb | 4060 |
| Phospho-p38 MAPK (Thr180/Tyr182) (D3F9) XP® Rabbit mAb | 4511 |
| Phospho-p44/42 MAPK (Erk1/2) (Thr202/Tyr204) (D13.14.4E) XP® Rabbit mAb | 4370 |
| Phospho-LAT (Tyr220) Antibody | 3584 |
| Phospho-Zap-70 (Tyr319)/Syk (Tyr352) (65E4) Rabbit mAb | 2717 |
| Phospho-SLP-76 (Ser376) (D9D6E) Rabbit mAb | 14745 |
| Phospho-Stat3 (Tyr705) (D3A7) XP® Rabbit mAb | 9145 |
| Phospho-Stat6 (Tyr641) Antibody | 9361 |
| Phospho-Stat2 (Tyr690) Antibody | 4441 |
| Phospho-Stat5 (Tyr694) (D47E7) XP® Rabbit mAb | 4322 |
| Phospho-c-Jun (Ser73) (D47G9) XP® Rabbit mAb | 3270 |
| Anti-rabbit IgG (H+L), F(ab')2 Fragment (PE Conjugate) | 79408 |
